# “We have a ticking time bomb”: a qualitative exploration of the impact of canine epilepsy on dog owners living in England

**DOI:** 10.1186/s12917-020-02669-w

**Published:** 2020-11-13

**Authors:** Amy E. Pergande, Zoe Belshaw, Holger A. Volk, Rowena M. A. Packer

**Affiliations:** 1 Clinical Science and Services, Royal Veterinary College, Hertfordshire, AL9 7TA UK; 2 PDSA Pet Hospital Nottingham, Dunkirk Road, Nottingham, NG7 2PH UK; 3 Small Animal Medicine and Surgery, University of Veterinary Medicine Hannover, Hannover, Germany

**Keywords:** Owner, Epilepsy, Seizure, Veterinary, Quality of life, Dog

## Abstract

**Background:**

Idiopathic epilepsy is a common neurological condition in dogs. Previous research has focused on clinical aspects of seizure management in dogs with idiopathic epilepsy with little attention given to the emotional and logistical challenges for their owners. The current study aimed to explore the impacts of owning a dog with idiopathic epilepsy on owner quality of life and lifestyle, using qualitative methods.

**Methods:**

Owners of dogs with idiopathic epilepsy living in England were recruited via social media and word of mouth, and then selected using purposive sampling to participate in face-to-face semi-structured interviews. Epilepsy management was explored through in-depth accounts of owner experiences and influencing factors. Interviews were recorded and transcribed verbatim and thematic analysis was used to construct key themes.

**Results:**

Twenty-one interviews were completed. Almost all owners had made lifestyle changes in order to care for their dog, including substantial modifications to routines and, in some cases, employment. Many owners discussed a very emotionally close dog-owner bond, and described experiencing frequent feelings of fear, stress and uncertainty regarding their dog’s health. Friends, family and colleagues did not always understand the magnitude of commitment required to care for a dog with idiopathic epilepsy. This, combined with a fear of leaving their dog unsupervised, had social implications in some instances and lead to increased use of the Internet and online groups for peer support.

**Conclusions:**

The commitment required to care for a dog with idiopathic epilepsy, and the lifestyle changes made by their owners, may be far greater than previously estimated. Further consideration of these factors by veterinary professionals and the friends and families of owners of dogs with idiopathic epilepsy could improve owner quality of life, and facilitate the provision of additional support.

**Supplementary Information:**

The online version contains supplementary material available at 10.1186/s12917-020-02669-w.

## Background

Epilepsy is a common chronic neurological condition in dogs [1], with an estimated prevalence of 0.62% in dogs presenting to primary practices in the United Kingdom (UK) [2]. Many dogs with recurrent seizures are diagnosed with idiopathic epilepsy, a term defined by the International Veterinary Epilepsy Task Force (IVETF) as genetic epilepsy, suspected genetic epilepsy, or epilepsy of an unknown cause [3]. A recent study found that 54% of dogs undergoing magnetic resonance imaging (MRI) for investigation of seizures had no structural lesions, and were considered to have idiopathic epilepsy [4]. Management of idiopathic epilepsy has typically focused on use of anti-seizure drugs to try and control the frequency and severity of seizures [5]. Despite potentially high monthly costs for epilepsy treatments [6] and the emotional and time commitments of owners to management, these efforts are often not proportional to success [7] and adequate seizure control may not be achieved [8, 9]. Historically, research on canine idiopathic epilepsy focused primarily on diagnosis and therapeutic management of the condition, but recently there has been increased awareness regarding the impacts on owners of dogs with idiopathic epilepsy [6, 10, 11].

In previous literature investigating owner quality of life, reference has been made to the World Health Organisation definition of health [12], which describes a “*state of complete physical, mental and social well-being, and not merely the absence of disease and infirmity*” [13, 14]. When discussing quality of life of pet owners, this definition provides an appropriate frame of reference when considering aspects of their life that could be impacted by caring for their pet. Research has shown that owners of chronically ill animals could detrimentally affect their own quality of life caring for their pet [15], and management of long term conditions can be challenging and distressing for owners [14]. Yet many owners choose to pursue advanced treatment options despite social and financial impacts [16], and place greater importance on their dog’s quality of life rather than their own lifestyle [17]. Owners of chronically ill animals may experience clinically significant levels of caregiver burden [18], increased stress, and signs of anxiety or depression [14]. This reflects human medical literature that describes parents of children with epilepsy experiencing higher rates of depression and anxiety, and a reduced quality of life, with significantly higher rates in parents of children with poorly controlled epilepsy versus those with well controlled epilepsy [19]. Informal caregivers, who provide voluntary care to human family members, can have high levels of responsibility and demand on personal time and energy [20]. Despite there being less existing research regarding caregiving for pets, there are evident parallels between the experiences of caring for people, and caring for chronically ill pets. In veterinary medicine, neurological conditions frequently present with clinical signs correlated with caregiver burden, which not only include seizures, but also signs relevant to medication side-effects and the post-ictal period, such as frequent urination or appearing depressed or anxious [21, 22]. Generalised seizures are physiologically stressful for owners to witness [11] and may cause more distress to the owner than the animal given the animal is unconscious while the seizures occur, although after regaining consciousness the post-ictal period could be potentially distressing to both parties especially if signs are prolonged [11, 22]. As the success of seizure control is not proportional to the commitment of the owner [7], it is also possible that owners may have feelings of helplessness [16].

To date, only survey-based studies have been used to explore owner quality of life in the specific context of canine epilepsy [6, 10]. Although these studies benefit from larger, sometimes international samples, applying qualitative methods to explore owner quality of life may offer greater insights into owners’ lives, and richer data on the effects of managing their dog upon their own quality of life. Previous qualitative studies of other chronic canine disorders e.g. osteoarthritis [23, 24] and behavioural problems [25], have produced detailed accounts of owner experiences using such methods, and identified novel impacts.

The aim of this study was to investigate the impacts of owning a dog with idiopathic epilepsy on owner quality of life and lifestyle, with particular consideration of potential emotional and social impacts. The objective was to perform semi-structured interviews and analyse their data thematically to explore owner experiences and attitudes in relation to managing a dog with canine idiopathic epilepsy.

## Results

### Participants and analysis

During recruitment, 155 prospective participants responded expressing interest in the study. Of these, 124 then completed the online screening questionnaire, and 62 met the inclusion criteria. Twenty-one face-to-face interviews were completed between October 2018 and May 2019 following selection using purposive sampling, and interviews ranged between 26 and 88 min in length.

Of the 21 respondents selected for interview, 15 were in the 40–59 year old age category, four were in the over 60 year old category, and two were in the 25–39 category. Interviews were conducted with the dog’s primary caregiver; however, if caregiving was shared equally more than one owner could be present at interview. Seventeen interviews were completed with one owner present (16 female and 1 male), and the other four were completed with two owners present (one same-sex couple and three opposite-sex couples). Nineteen interviews were conducted at owners’ homes, one at the Royal Veterinary College, UK, and one at a neutral public location. The participants were recruited from 13 different counties in England. The most frequently represented dog breeds were Springer Spaniels (*n* = 3), Cross-breeds (*n* = 3), and Border Collies (*n* = 2), with all other breeds included being represented once only. All but one of the included dogs were being treated with prescription anti-seizure drugs, and 12 of the 21 owners reported use of adjunctive treatments in the pre-interview questionnaire. Fourteen of the dogs included had been referred for assessment by a neurologist, or MRI, and dogs with both generalised seizures and focal seizures were represented.

The majority of codes were defined in the analysis of the first 12 interviews, and then identification of codes slowed. However, in the fifteenth and sixteenth interviews a number of new codes were created that added greater insight to the themes being formed. It was felt that at the end of the 21 interviews both code and meaning saturation had been achieved. From the data collected, four themes were formed during the analysis to describe owner experiences and factors contributing to decision-making. In this paper the theme ‘impacts on owners – “we have a ticking time bomb”’ will be reported, using illustrative quotes to exemplify findings. Quotes have been shortened or contextualised where necessary, denoted by ‘[…]’, but all original meaning is respected and maintained.

### Theme: impacts on owners – “we have a ticking time bomb”

#### Distress relating to seizures

Owning a dog with idiopathic epilepsy had a significant impact on the quality of life of owners, and owner lifestyle. Following their dog’s initial seizure all interviewees recalled feeling negative emotions such as being distraught, fearful and uncertain regarding disease progression. Prior experience with canine epilepsy was rare, and owners were shocked and distressed by the appearance of the seizures.

*I was just distraught when we first found out* [he had epilepsy]*, didn’t want to leave him at all, I wouldn’t even go to the shop.* [Interview 4]

*When* [the first seizure] *happened, we were devastated,* [my husband] *just thought he was dead when he fell off the bed…* [Interview 9]

Seizures were very distressing to witness, and often this was intensified due to the unpredictable nature of idiopathic epilepsy e.g. when and how often seizures occur. Owners searched for prodromal signs (pre-seizure behavioural changes) or seizure triggers to try to anticipate when seizures were coming, which they felt might give them a greater sense of control. Owners described the unpredictable and sometimes inconvenient timing of their dog’s seizures, with some participants discussing impacts on sleep and wellbeing as a result.

*It has an impact on my sleep sometimes, when it’s late at night and she wants me to stay down here after she’s had the fit and I’ve got to get up for work in the morning, I think that’s the, it’s the timing of them is the biggest impact.* [Interview 16]

*I think we just need to get used to it a bit more, because we always feel a bit, like I said before, like we’re living with a ticking time bomb.* [Interview 6]

Most participants kept records of their dog’s epilepsy. Often this had initially been recommended by their veterinary surgeon, but owners included greater detail than typically requested such as medication doses, adjunctive therapies, laboratory test results, and the lunar calendar; the latter due to beliefs it affected the likelihood of a seizure occurring. This dedication and attention to detail seemed again to relate to the unpredictability of the seizures, and was linked to a search for patterns in their dog’s seizure activity. Often owners spoke positively about keeping these records, due to the potential to use them to benefit the dog’s care, and they appeared to be a beneficial tool not only for veterinary surgeons, but also for owners to monitor their dog’s progress at home and search for possible triggers.

*…so when you get the spreadsheet, there’s all the different dates and the different levels and everything. So, yes, so you’ve got one for all her fits, blood results and supplements and things that she’s on…* [Interview 16]

#### Lifestyle changes

Many interviewees acknowledged that their quality of life was reduced in some way by their dog’s epilepsy. Other owners reported experiencing lifestyle changes, but avoided directly describing their quality of life as being affected. However, the changes described often appeared to involve significant effort, commitment and lifestyle restriction. Often limitations related to strict medication schedules (with dogs requiring drug administration twice or three times per day at set times), difficulty finding assistance in caring for the dog, and an inability to leave the house for leisure activities or vacations. Owners frequently stated that they did not mind making these adjustments for their pet, usually highlighting the close emotional bond between themselves and their dog, but sometimes citing other emotional reasons such as guilt. Some participants also reported that their employment had changed due to their dog’s idiopathic epilepsy. Some owners had reduced their working hours or started working from home, whereas others had ceased working entirely to care for their dog.

*Well* [my quality of life is] *pretty bad now* […] *I can’t go out and get a proper job anymore, I’m not really earning much money from doing this* [freelance work] *but I’m doing this because I have to be around, I’m doing everything for him but I’m not really earning a living…* [Interview 3]

*Yes, I’ve changed, I don’t go on holiday unless she can come, I changed the way my routine* [is]*, I’ve changed all the things I can do, that’s definitely affected things, but it’s alright, I don’t mind, it’s not her fault she’s got it and it’s my fault I bought her from a dodgy breeder.* [Interview 11]

Contrastingly, a couple of participants discussed that caring for their dogs did not significantly impact their day-to-day life or emotions anymore. This represented a small minority of participants and was linked to seizure control. This finding was primarily discussed by an interviewee whose dog had not had a seizure for a number of years.

*I think now because we’ve got the epilepsy under control, I’m just touching wood when I say this, we don’t actually day to day tend to think about the epilepsy too much.* [Interview 5]

#### The dog-owner bond

The vast majority of participants discussed having a strong dog-owner bond. Some thought that this bond was strengthened due to their dog having epilepsy, and the associated commitments they had made to care for them. Sometimes the relationship was likened to those with human family members, and several owners reflected that their relationship with their dog with epilepsy was closer, or in other ways different, compared to those with other dogs they currently or previously owned.

*I mean we were always completely inseparable anyway, as I say, he’s my baby, he’s my absolutely everything, but yeah, now he’s just my poorly baby.* [Interview 4]

*I mean, maybe,* [my dog with epilepsy] *might be my favourite but don’t tell* [my other dog][laughs], *shh. Only because of this, he’s been through quite a lot…* [Interview 18]

No participants described having a less emotionally close relationship due to their dog having idiopathic epilepsy, although a couple of interviewees reported that the impacts of caring for a dog with idiopathic epilepsy potentially reduced the enjoyment they got from owning their dog.

*I suppose I can’t, it sounds awful, but I can’t enjoy him as much as I used to, but I think that’s my anxiety.* [Interview 10]

This bond also caused owners to become more involved in decision-making regarding their dog’s veterinary care in a number of cases. Due to the close dog-owner bond and stress regarding their dog’s future, owners often felt that they needed to be a strong spokesperson for their dog when discussing their healthcare. Some owners felt that they were more able to contribute to discussions with their veterinary surgeon regarding treatments and on-going management due to having a better understanding than their veterinary surgeon of the care involved in managing their dog at home.

*Perhaps some people won’t be confident enough to do that but I know my dog very well and I think that I’m probably the best-qualified person to decide what is best for him.* [Interview 1]

Owners were commonly frustrated and disappointed with lack of treatment success and on-going medication side effects, and expressed feelings of helplessness. Sometimes this was again linked to the dog-owner relationship, for instance in the case of side effects where owners felt their dog’s demeanour or personality was affected. However, in other instances it was linked to lifestyle-related or financial issues rather than being a purely emotional impact. In a number of cases these feelings of helplessness were also associated with despair, where owners had lost hope of their dog’s condition improving.

*I think it’s like when you have a child, you’re helpless. If something happens to them you want to take that pain away, and nothing you can do, despite knowing what’s happening, you feel, it’s that helplessness.* [Interview 7]

*It was sad, it was really upsetting, because he really, he changed so much with the drugs as well, he just, he was a different dog.* [Interview 18]

#### Social impacts

The majority of interviewees reported that other people external to the household struggled to understand the lifestyle, emotional and logistical impacts of owning a dog with epilepsy. This lack of understanding sometimes caused feelings of isolation, and was one of the most significant contributors to a reduced quality of life for some owners. Due to the episodic nature of idiopathic epilepsy, and many dogs appearing normal between seizures, owners often believed that other people with no prior experience of the condition could not appreciate the potential impact of the seizures, and were not understanding of the owner’s commitments. Feelings of isolation were exacerbated where owners cancelled or avoided social events due to fear of leaving their dog.

…*I fret and stress and everything and I could see my friend getting really exasperated with me, but that night he had a seizure in front of her and she just burst into tears and it just changed her view entirely*… [Interview 4]

*…I’ll cancel events, and I have done many times, you know, family things and that where he’s just not quite seemed right.* [Interview 21]

Fear of judgement from others and feelings of helplessness also appeared to increase use of the Internet in some instances. Many participants discussed the use of online groups or social media pages as a source of additional support, with a number going on to discuss the community aspects both positively and negatively. There were some perceived benefits, including peer-peer emotional support and the availability of anecdotal evidence regarding medication side effects and novel management methods. However, some owners felt accessing these groups just added to their stress and was a source of negativity, with discussions of drug-refractory cases or emergency situations (e.g. status epilepticus; prolonged, uncontrollable seizures) adding to their fears for their own dog.

*…there’s people that are on those forums and they’re new to it, and their world has just ended, they love their dog to bits, and they’ve had a seizure and they can’t cope, and to see them in that state, they’re so distraught…* [Interview 7]

*The forums I found a bit scary and overwhelming, because it was clearly full of people that had even less of an idea than me…* [Interview 4]

Overall, having a dog with idiopathic epilepsy had a number of significant impacts on the lives of owners. Many owners acknowledged that their own quality of life had deteriorated, or at the very least their lifestyle was different compared to before their dog’s diagnosis. This was not the case for every participant, and the impact on quality of life appeared related to the dog’s seizure frequency and the extent of the emotional benefits reaped from the dog-owner bond.

## Discussion

This study provides new insights into the concerns and experiences of owners of dogs with idiopathic epilepsy, and the impact of caring for a dog with epilepsy on day-to-day routine and lifestyle. The use of a qualitative methodology allowed influencing factors and emotional aspects to be explored in depth. A number of direct impacts on owner quality of life were identified, including emotional and financial factors and the time associated with providing care for their dog, as well as revealing secondary impacts such as lack of sleep and social isolation. These findings illustrate for the first time the extent of the challenges that owners of dogs with idiopathic epilepsy might experience.

The impact experienced by owners was greater than anticipated by the investigators, although elements relating to stress, limitations and difficulty sleeping have been noted in previous literature regarding animals with chronic illnesses [16, 17], and parents of children with epilepsy [26]. However, this study provides greater understanding of areas of importance with regards to canine idiopathic epilepsy specifically, and also highlights the complexity of the issues experienced by owners. Previous research found that owners of chronically ill animals can feel helplessness when clinical signs are not controlled, and this seems common in owners of dogs with idiopathic epilepsy when seizures are not adequately controlled by anti-seizure drugs [16]. However, another recent study found that composite scores for quality of life of dogs with epilepsy and their owners were generally positive [6]. These findings could relate to an owner’s interpretation of the definition of quality of life, or the occurrence of normalisation phenomena; indeed, even owners who did not report that their quality of life was negatively affected by epilepsy management still described significant changes in their own behaviour patterns, livelihoods and social lives. It is possible that owner evaluation of quality of life may be impacted by the extremely close dog-owner bond noted, and some owners did not feel their quality of life was reduced due to them being willing caregivers who psychologically benefit in other ways from owning their dog, despite it’s epilepsy. This is supported by previous findings that owners of epileptic dogs are more concerned with their dog’s quality of life and seizure frequency rather than impacts on their own finances and lifestyle [17]. It has also been recommended that caregiving impacts experienced by parents of children with epilepsy should not be inferred as positive or negative, as depending on the views of the parent and the context many impacts could be deemed as either, or have both positive and negative outcomes [26]. This is also likely to be the case for impacts experienced by owners of dogs with idiopathic epilepsy.

An intense emotional attachment can result in greater motivation to pursue further treatments [27], and owners may feel more obliged to provide care for their chronically ill pet [16]. The dog-owner bond could also be influenced by the presence of neurobehavioural comorbidities. Dogs with idiopathic epilepsy can demonstrate increased fear and anxiety [28, 29], and owners of dogs with increased social fear have previously been found to perceive their relationship with their dog to be closer than owners of less fearful dogs [30]. Similar findings were also reported in a recent qualitative study regarding pets with behavioural problems, where a close pet-owner bond was noted by some owners despite them feeling a variety of negative emotions such as stress, fear and anxiety as a result of their pet’s problem [25]. A very emotionally close relationship could predispose owners to have greater feelings of uncertainty, anxiety and despair regarding their dog’s future, and increase caregiver burden. This seemed to be reflected by the degree of dedication, and meticulous management and record keeping demonstrated by some owners. Strict routine seemed to reduce feelings of uncertainty, and perhaps also benefit the few owners who had expressed feelings of guilt. This has parallels to previous research finding family routines contributed to greater feelings of competency and satisfaction in parents [31].

The emotional impacts experienced by the participants may indicate a need for increased support for owners of dogs with epilepsy. Potential challenges and negative outcomes may not be adequately discussed by veterinary surgeons in an attempt to not upset or overwhelm owners, or due to lack of awareness of these challenges. However, avoiding the provision of data on negative outcomes in epilepsy may not necessarily be beneficial to carer quality of life. In human medicine, the provision of information by paediatric neurologists regarding sudden death in children with epilepsy has been discussed with parents, and there were concerns that it could be unnecessarily shocking or stressful for parents [32]. However, despite these concerns, 91% of parents in the same study wanted to be provided with the information [32]. Therefore, there may be some benefit in veterinary surgeons discussing more severe clinical signs or outcomes in canine epilepsy with dog owners, but this would need to be assessed on an individual basis, and further research is required to identify what information owners would find valuable, and at what stage of their dog’s condition.

It has previously been found that owners who were uncertain or concerned regarding recommendations from their veterinary surgeon seemed more likely to use the Internet to perform self-directed research [33]. Due to interviewees expressing feelings of stress and uncertainty regarding their dog’s disease process, some owners of dogs with idiopathic epilepsy may also be more likely to use the Internet, which could be problematic due to misinformation. The use of online groups was controversial amongst participants, and whilst some valued emotional support and anecdotes, others were overwhelmed by perceived negativity. The creation of a moderated environment for owners to use to communicate online with peers could reduce fear and improve satisfaction while ensuring accurate medical information is provided. Indeed, in human medicine, technological advancements have facilitated novel approaches to supporting caregivers, for example the use of videoconferencing for isolated caregivers [34], and such technologies could be explored in veterinary medicine. Increased social support has been found to be associated with lower parenting stress in parents of children with epilepsy [35], so facilitating positive interactions between owners could improve owner quality of life and reduce feelings of stress or isolation. There may be an increasing need for veterinary social work [18] and use of tools to assess caregiver burden and create open conversation [21]. Taking this into consideration for the future, greater awareness and empathy from veterinary surgeons and the families of owners could lead to improved emotional support, and in conjunction with more targeted resources and communication platforms this could improve their experience of caring for their dog.

There are some limitations associated with this study. There may be bias due to owners needing to volunteer for participation, and therefore these findings may only represent the views of more involved owners likely to undertake research activities, and so should not be extrapolated to all owners of dogs with idiopathic epilepsy. However, the views of this subset of owners are valuable, and give further understanding of the challenges owners might face and have substantially added to understanding above and beyond the findings of previous quantitative studies in this area. Equally, the majority of participants in this study were female, which could be due to women being more willing to participate or viewing themselves as having a more significant caregiving role, but this high proportion of female respondents has also been noted in previous relevant studies [16, 18, 25, 36]. Another limitation was that the diagnosis of idiopathic epilepsy was reliant on owner reporting. However, the included dogs all had clinical presentations consistent with idiopathic epilepsy, and as this study addresses owner impacts rather than clinical variables, the inclusion criteria was sufficient to ensure an accurate representation of owner experiences. Despite these limitations, this is the first time qualitative research has been used to explore this topic, and this project has highlighted previously unrecognised insights which can now be investigated further and assist in producing improved resources for owners. A larger scale international survey based on these qualitative findings is underway to investigate these aspects in more detail.

## Conclusions

These findings increase our understanding of the sometimes negative and often profound emotional effects and lifestyle changes experienced by owners managing a dog with idiopathic epilepsy. Direct impacts such as those to finances and time, and indirect impacts such as social isolation and sleep deprivation, were evident. The significance of these impacts appeared to be connected to the dog’s seizure frequency. Due to a close dog-owner bond, owners were typically willing to make these changes in order to care for their dog. With better support and resources, these impacts could potentially be minimised to protect owner quality of life.

## Methods

Ethical approval for this research was granted by the Royal Veterinary College Social Science Research Ethical Review Board, UK (reference SR2018–1625). This significant piece of research was designed to broadly investigate owner decision-making and experiences of owning a dog with epilepsy. This report discusses the findings regarding owner quality of life relevant to the specific study aims.

### Inclusion criteria

Inclusion criteria for interviewees were: a) ownership of a dog diagnosed with idiopathic epilepsy, b) residency in the UK, and c) availability for interview during the study period. The diagnosis of idiopathic epilepsy had to be consistent with a Tier I confidence level, as previously defined by the IVETF [37], or made after referral and MRI showing no structural lesions. A Tier I confidence level includes a history of more than one seizure at least 48 h apart, an age of 6 months to 6 years at time of first seizure, and no cause found on physical examination, neurological examination, or blood and urine testing [37]. This was established from the date of first seizure and the diagnostic tests performed for each dog, as reported by owners in the pre-interview screening questionnaire. If these criteria were not met subjects were excluded from participation. Dogs with medical comorbidities were permitted to participate in the study, unless the comorbidity was likely to be relevant to the dog’s seizures e.g. a history of inflammatory brain disease or head trauma, in which case they were also excluded. This was assessed on an individual basis by a qualified veterinary surgeon (AEP) using the information provided.

### Recruitment

Owners of dogs with idiopathic epilepsy were recruited from August 2018 until data saturation was reached via distribution of a study poster on social media, and word of mouth through veterinary practices and other research activities. The poster was widely shared across canine epilepsy pages, breed-specific pages, and veterinary pages on social media, and was viewed by over 76,000 people on Facebook alone. Prospective participants were then required to complete a short online screening questionnaire to collect basic information about the owner and their dog, and to ensure the inclusion criteria were met. This included age, sex, diagnostic tests performed, management to date and geographical location. Purposive sampling, which is a method frequently utilised in qualitative research, was used to select information-rich cases to provide new insights regarding the research question [38, 39]. Primarily, variation was aimed for with regards to the number and type of medical and adjunctive treatments used, and whether referral to a specialist neurologist had been made. Consideration was also given to ensure owners of different sexes, age groups and geographical locations were represented.

### Owner interviews

The interview schedule (see Supplementary File 1) was constructed by the authors and was influenced by previous qualitative studies focusing on caregiver burden in owners and chronic diseases in companion animals [16, 23]. Prior to commencing the interviews, participants were provided with supplementary information regarding the study and were required to read and sign the study consent form, granting consent for interview and for anonymised data to be published. Two pilot interviews were completed and transcribed by a professional external transcription company in intelligent verbatim before commencing data collection. These pilot interviews were conducted in August and September 2018, lasting 38 and 42 min respectively, and were not included in the thematic analysis. After reviewing these pilot transcripts, the breadth and content of the interview data was deemed fit for purpose and the interview schedule was not amended. Interviews were conducted at the owner’s home or a neutral location of their choice by one researcher (AEP), and were recorded using a Dictaphone before being transcribed.

### Thematic analysis

Thematic analysis is a fundamental qualitative method capable of providing a complete and involved account of interview data [40]. The process of completing a thematic analysis comprises of locating and recording patterns of meaning, also known as ‘themes’, and has previously been described in detail [40]. The six-step method of thematic analysis described by Braun and Clarke (2006) was followed, which involves familiarisation with the data, generation of codes, creating themes, reviewing the themes, defining and naming the themes and producing a written report [40]. Coding is a method used to identify basic elements of the data, and allows segments to be organised prior to creating themes which are an analytic product representing shared patterns of meaning [40, 41]. There is minimal consensus regarding data saturation in qualitative research, but previous literature describes a significant difference between the number of interviews required for code saturation (identification of thematic issues) versus meaning saturation (a rich understanding of thematic issues) [42]. In this study the aim was to reach meaning saturation, and this was achieved by reflexive evaluation involving continuous reflection and engagement with the data [41]. Formal training in the analysis of qualitative interviews was obtained by AEP from the Health Experiences Research Group at the University of Oxford, UK. Each transcript was manually coded on paper at least twice during the analytic process. Details of the different codes and their location in the data were documented, and as the analysis progressed codes were reviewed and categorised as deemed appropriate. The ‘One Sheet of Paper’ (OSOP) method [43] was utilised to enhance understanding of patterns within the data, and allow visual mapping of codes to create themes. The analysis was not reviewed by the participants; however, the interview transcripts and recordings were frequently reassessed to preserve the context and owners’ meaning.

This research was completed using the contextualist method of critical realism, allowing a true representation of participants’ realities whilst accounting for context and the potential influence of social constructs [40]. Therefore, the analysis performed aimed to represent the true feelings and experiences of the owners included, whilst exploring how these could impact future decisions. Alongside data collection, thematic analysis was performed as an iterative process where small groups of interviews were analysed at one time. This ensured important areas of focus could be recognised, and potential themes formed, whilst identifying when data saturation was being approached.

## Supplementary Information

**Additional file 1:** **Supplementary File 1.** Includes the interview schedule and prompts developed for this study, which were used as the basis for semi-structured interviews with participants.

## Data Availability

The datasets generated and/or analysed during the current study are not publicly available due to the potential to compromise participant consent or confidentiality, but are available from the corresponding author on reasonable request.

## Abbreviations

IVETF: International Veterinary Epilepsy Task Force
MRI: Magnetic Resonance Imaging
UK: United Kingdom
