# Supplementary material for: “We have a ticking time bomb”: a qualitative exploration of the impact of canine epilepsy on dog owners living in England
Source: BMC Vet Res. 2020 Nov 13;16:443. doi: 10.1186/s12917-020-02669-w (PMC7666515; doi:10.1186/s12917-020-02669-w)
Supplement: Supplementary file 1 — Additional file 1: Supplementary File 1. Includes the interview schedule and prompts developed for this study, which were used as the basis for semi-structured interviews with participants. [file 12917_2020_2669_MOESM1_ESM.docx]

**Interview Schedule**

1. Tell me about [dog’s name] and [his/her] general background, in terms of when you got [him/her] and any previous health concerns.
   1. When did you get [him/her], and where from? What age was [he/she]?
   2. Any health problems other than epilepsy? If so what are these and when were they diagnosed?
   3. Previous dog owning experience – same breed? Other dogs/pets in the house currently? Any previous dogs with chronic health problems?
   4. Do you have any other prior experience with epilepsy, either in dogs or humans?
2. Now we will talk about the diagnosis of idiopathic epilepsy in [dog’s name], tell me about when [he/she] started having seizures and what happened next.
   1. First seizure – seizure type, when/where, what happened afterwards?
   2. When was the diagnosis of epilepsy made?
   3. What tests were performed to reach this diagnosis?
   4. Did your vet explain how the diagnosis was reached? What information was given to you by your vet?
   5. How did you feel after the diagnosis? Did you feel you understood the condition and treatments available?
   6. What does epilepsy mean to you/how do you describe it to your friends?
   7. What were your expectations on seizure control/what were you told?
   8. How did you decide on the initial treatment plan?
   9. Were you given any information about alternative treatment options? Was this useful?
3. Now that we know how the diagnosis was made, we’ll discuss management. Is [dog’s name] currently on any treatments? Please describe what the management currently involves.
   1. What treatments or therapies are you currently using to treat your dog’s epilepsy?
   2. How do you feel about [dog’s name] being on treatment? Reducing seizures v. risk of side effects?
   3. Do you feel that this treatment satisfactorily reduces the seizure frequency/severity? How does this fit with your initial expectations?
   4. Is this different to what you have used previously? If so please describe what made you change.
   5. Did your vet guide you to decide on the current management? If not, what other information sources did you use and how did you hear about them?
   6. Do you have any concerns regarding side effects? Either with regards to current or previous treatments used.
   7. How often do you alter your management/medication schedule? Do you always consult your vet prior to doing so? Do you have trouble administering the medication or remembering doses? Do you do anything else other than what your vet advised?
   8. Do you use any alternative therapies, e.g. supplements or homeopathy? Did your vet tell you about these therapies?
   9. Do you have any plans to alter the regime in the future, or are there any new treatments you currently want to try?
   10. How do you monitor your dog’s epilepsy? Do you go for regular rechecks with your vet, if so do you find them useful?
   11. Have you noticed any changes in your dog aside from the fits – either in behaviour, trainability or attitude?
   12. Where do you find support regarding your dog’s epilepsy? E.g. friends, family, forums, strangers.
   13. Has there been a financial impact?
4. Tell me more about how you make decisions regarding your dog’s epilepsy.
   1. Decision making regarding new treatments or changes – on-going involvement of the vet?
   2. Where do you get your advice from/where will you get your advice from in the future?
   3. Involvement of family/friends in decision-making?
   4. Do you think your thoughts on other medical conditions have changed since your dog started having seizures? Are you more or less likely to go to the vets?
   5. Have you gathered/do you share information via any other sources since the diagnosis? E.g. friends, the Internet…
   6. Are you involved with/aware of any forums or support groups?
5. We’re now going to discuss quality of life and your relationship with your dog, do you think either has changed as a result of [him/her] having epilepsy?
   1. How do you judge your dog’s quality of life? Do you think it has changed since [he/she] started having seizures?
   2. Do you feel that your relationship with your dog has changed since the diagnosis? If so how?
   3. If you’ve owned dogs previously, do you feel your relationship is different with [dog’s name] due to [him/her] having epilepsy?
   4. Do you find you worry more about [dog’s name]? It has been suggested that owners of dogs with chronic diseases may feel more worried and like they are a carer – do you agree with this?
   5. Other owners have been of the opinion that their dog’s epilepsy affects their quality of life, how do you feel about this? How do you feel about epilepsy and seizures compared to at the initial diagnosis?
   6. Do you think you and your vet perceive your dog’s quality of life differently?
6. Is there anything else you would like to discuss that hasn’t been covered?
